# A case of severe anaphylactic reaction after administration of diagnostic-dose ferumoxytol in a pediatric patient

**DOI:** 10.1007/s00247-024-05970-0

**Published:** 2024-06-22

**Authors:** Fam Ekladious, David Saul

**Affiliations:** 1 Thomas Jefferson University Hospital, 132 S 10th St, Suite 780-K Main, Philadelphia, PA 19107 USA; 2 Nemours Children’s Hospital, Delaware, 1600 Rockland Road, Wilmington, DE 19803 USA

**Keywords:** Anaphylactic reaction, Ferumoxytol, Pediatric, MRI

## Abstract

We describe a case of anaphylaxis during administration of intravenous (IV) ferumoxytol as a magnetic resonance imaging (MRI) contrast agent in a 4-year-old patient with complicated past medical history including YARS genetic mutation with resultant liver failure and deceased donor liver transplantation, stage IV chronic kidney disease (CKD), and hypertension. The patient was noted to have labored breathing 4 min after initiation of ferumoxytol infusion and was subsequently rapidly intubated and returned to the intensive care unit (ICU) for monitoring. Anaphylactic reactions to therapeutic doses of ferumoxytol led to issuance of a black box warning by the FDA in 2015. Adverse reactions to lower-dose ferumoxytol used in diagnostic imaging, however, are rare and there has been a paucity of documented anaphylactic reactions in the literature.

## Introduction

Ferumoxytol, a supermagnetic iron oxide nanoparticle (SPIO), has unique imaging properties including a long intravascular half-life of 15 h. Additionally, there is no known risk of nephrogenic systemic fibrosis (NSF) with its administration. A black box warning issued for ferumoxytol in 2015 due to acute hypersensitivity reactions in patients who received therapeutic dose ferumoxytol, including 79 reported anaphylactic reactions and 18 fatalities, has led to hesitancy in the utilization of lower-dose ferumoxytol as a contrast agent. Multiple retrospective studies and systemic reviews reveal, however, only one documented case of possible anaphylactic reaction after administration of ferumoxytol as a contrast agent [1].

## Case report

The patient is a 4-year-old girl with YARS genetic mutation and consequent liver failure and chronic kidney disease. The patient underwent liver transplantation during her admission which was complicated by anemia (requiring exploratory laparotomy), lymphatic leak, and medically resistant hypertension. A magnetic resonance angiography (MRA) of the abdomen for assessment of the renal vasculature was scheduled as part of the workup of her persistent hypertension. Slow infusion of 0.06 mg/s of 57 mg (4 mg/kg) of ferumoxytol distilled in 25 mL of saline was initiated on the day of the scheduled MR, prior to scheduled general anesthesia and before the patient was brought for imaging. The patient’s baseline vitals prior to initiation of the infusion were a heartrate of 105 beats per minute (BPM), a respiratory rate of 24, and a blood pressure of 122/84 mmHg. Approximately 4 min after initiation of infusion, the patient was noted by the mother and medical staff to have labored and shallow breathing with decreased breath sounds. Physical exam also demonstrated weak pulses and decreased capillary refill with increased skin pallor, prompting cessation of the ferumoxytol infusion and initiation of a code. At this time point, the patient had received approximately 15 mg of ferumoxytol over 4 min since initiation of the infusion. The patient was bagged and masked and subsequently intubated. Vital signs were taken at the time of anaphylaxis and regularly thereafter with a documented blood pressure of 48/21 mmHg, a heart rate of 116 BPM, and a respiratory rate of 19 approximately 11 min after infusion initiation, after the patient had been intubated. Resuscitation was initiated with dextrose 5% and 0.9% sodium chloride for a total volume of 1,000 milliliters (mL). The patient was initiated on an epinephrine drip at a rate of 0.05 mcg/kg/min with blood pressure eventually normalizing. Anti-histamines and steroids were also administered at the time. The MR examination was canceled and the patient was returned to the ICU for monitoring. The patient was extubated the following day.

Our patient met one of the diagnostic criteria for anaphylaxis as defined by the World Allergy Organization as “acute onset of hypotension or bronchospasm or laryngeal involvement after exposure to a known or highly probable allergen…even in the absence of typical skin involvement” [2]. Additionally, lab-work drawn during the code further supported the diagnosis of anaphylaxis, revealing respiratory acidemia with a venous pH of 7.13, decreased from a baseline of 7.3-7.5 measured in the weeks prior.

## Discussion

Gadolinium-based contrast agents (GBCAs) are the most utilized contrast agents in MR imaging but have potential adverse events including risk for nephrogenic systemic fibrosis (NSF) and deposition in neuronal tissues [3]. Thus, radiologists have sought alternative contrast agents for utilization in vulnerable populations such as in patients with renal failure. While the imaging properties of GBCAs and ferumoxytol are distinct, ferumoxytol can be an attractive alternative contrast agent in specific clinical contexts. Particularly, ferumoxytol has no known risk of NSF due to its large particle size of 30 nanometers (nm) which prohibits its glomerular filtration. Ferumoxytol is instead taken up in the reticuloendothelial system by macrophages and astrocytes where its carboxymethyldextran coating is cleaved in secondary lysosomes; the iron core is then incorporated into the body’s iron stores or used for hemoglobin synthesis [4]. Further, ferumoxytol has the added benefit of a long intravascular half-life of 15 h, affording practical clinical benefits such as increased time window for image acquisition (particularly useful in the pediatric population) and improved image quality of MR angiography and venography examinations when compared to GBCAs [5]. Ferumoxytol, in contradistinction to GBCAs, can provide negative contrast imaging through T2* shortening properties, a property which has been utilized for improved detection of occult malignant lymph node metastasis, imaging of tumor necrosis, and diagnosis of osteomyelitis [6].

Ferumoxytol, initially developed for utilization as an MR contrast agent, gained FDA approval for therapeutic treatment of iron deficiency anemia in adults with chronic kidney disease. A black box warning was issued by the FDA in 2015 due to concerns for acute hypersensitivity reactions, with 79 reported anaphylactic reactions and 18 fatalities in approximately 1.2 million administrations [7]. Multiple retrospective studies and a few prospective studies sought to analyze the incidence of adverse events after utilization of diagnostic-dose ferumoxytol, which is typically administered at a dose of 1-7.5 mg/kg for a maximum total dose of 510 mg (compared to a typical therapeutic dosage of 1,020 mg divided over two administrations) diluted and slowly infused over 15 min. To date, no definite anaphylactic reactions to diagnostic doses of ferumoxytol, utilizing diagnostic criteria delineated by the World Allergy Organization, have been documented in these analyses. A systematic review by Ahmad et al. reported an adverse event rate of off-label ferumoxytol use of 2% [1]. Of note, one possible anaphylactic reaction was documented in a retrospective analysis by Wise-Faberowski et al., wherein a patient developed hypotension (requiring vasopressors and intubation) without skin manifestations or respiratory complications; the authors suggested that the reaction may have been related to sedation rather than ferumoxytol [8].

Our patient had convincing evidence of anaphylaxis after infusion of diagnostic-dose ferumoxytol with respiratory function deteriorating minutes after initiation of the infusion, ultimately leading to intubation. Further, our patient developed shock with hypotension requiring administration of epinephrine and anti-histamines. Lab-work drawn at the time of the code confirmed respiratory acidosis and a chest radiograph demonstrated new pleural effusion with vascular congestion.

Our patient’s medical comorbidities presumably placed her at higher risk for an adverse event and thus the patient was assessed by an anesthesiologist prior to her scan, per our usual protocol for medically complex patients or those who require general anesthesia for developmental/airway issues. Additionally, her medical issues were considered as per our usual hospital protocol, which entails induction under the supervision of an anesthesia provider and nursing staff with monitoring per standard hospital policy. As with all patients at risk for anaphylaxis, she had a full order set for PRN anaphylaxis medications ordered prior to initiation of the infusion.

Ferumoxytol still remains an attractive MR contrast agent, particularly when there is concern for nephrogenic systemic fibrosis, and provides a range of unique imaging applications relative to GBCAs. While the FDA black box warning states that monitoring of patients during ferumoxytol infusion is recommended as standard practice, the rate of severe reaction for diagnostic-dose usage is extremely low and the true anaphylactic reaction rate is unknown. As recommended by the FDA, our case supports the need for continuous clinical and hemodynamic monitoring when administering ferumoxytol for diagnostic purposes. Heightened awareness and risk-benefit analyses should be taken into consideration particularly in those patients with complex medical histories and history of multiple atopies/allergies.
